# 

**Published:** 1906-09-29

**Authors:** 


					the hospital.
A
Sept. 29th, 1906. .
/
OSE ITALf
_ ??? i hoscuoma
LAscorr IVe^eru iNnKuAM ^   ?
No. ] fy^
KT  f~" INFIRMARY
Vjol. XL. [SS.-] Saturday,
Sept. 29th, 1906.
1 ?' ^NiiMblX^'^MmcSTSSStSr^5'
PRICE THREEPENCE

				

